# Optimising Exposure for Children and Adolescents with Anxiety, OCD and PTSD: A Systematic Review

**DOI:** 10.1007/s10567-020-00335-z

**Published:** 2021-02-06

**Authors:** Hannah Plaisted, Polly Waite, Kate Gordon, Cathy Creswell

**Affiliations:** 1 School of Psychology, University of Birmingham, Birmingham, UK; 2 School of Psychology and Clinical Language Sciences, University of Reading, Reading, UK; 3 Department of Experimental Psychology & Department of Psychiatry, University of Oxford, Oxford, UK; 4 Berkshire Healthcare NHS Foundation Trust, Berkshire, UK

**Keywords:** Child, Adolescent, Youth, Anxiety, Exposure, Cognitive behaviour therapy

## Abstract

Cognitive behavioural therapy is an effective treatment for anxiety disorders in children and young people; however, many do not benefit. Behavioural exposure appears to be the critical ingredient in the treatment of anxiety disorders. Research with adults has identified innovative strategies to optimise exposure-based treatments, yet it is not clear how to optimise the effects of exposure for children and young people. This review was a preliminary exploration of the association between potential optimisation strategies and treatment procedures and outcomes for the treatment of child anxiety symptoms/disorders. We searched Psych-Info and Medline databases using a systematic search strategy and identified 29 articles. We found preliminary evidence that some specific strategies may enhance the effects of exposure, such as dropping safety behaviours, parents and therapists discouraging avoidance, and the use of homework. However, not one significant finding was replicated by another study for the same timepoint using the same methodology. To a large degree, this lack of replication reflects a limited number of studies combined with a lack of consistency across studies around conceptualisations, methodological approaches, and outcome measures making it difficult to make meaningful comparisons between studies and draw firm conclusions. Examination is needed of a wide range of theoretically-driven potential optimisation strategies using methodologically robust, preclinical studies with children and young people. Furthermore, the methods used in future research must enable comparisons across studies and explore developmental differences in the effects of particular optimisation strategies.

## Introduction

Anxiety and related disorders^1^ are among the most common and impairing mental health disorders in children and adolescents, with worldwide prevalence rates estimated at 6.5% (Polanczyk et al. 2015). This is of particular concern as, if left untreated, childhood anxiety disorders can run a chronic course (Bittner et al. 2007; Broeren et al. 2013), are associated with educational underachievement (Owens et al. 2012), poor peer relationships (Asendorpf et al. 2008) and poor social (Settipani and Kendall 2013) and occupational (Swan and Kendall 2016) functioning.

Cognitive behavioural therapy (CBT) is typically the first-line treatment for child and adolescent anxiety disorders (e.g. National Institute for Health and Care Excellence 2014; World Health Organization 2015). Although CBT is an effective treatment, approximately 41% of young people do not benefit (James et al. 2015). Furthermore, a naturalistic follow-up study of anxious youth treated with CBT found that 48% of initial treatment responders relapsed following treatment (≤ 6 years) (Ginsburg et al. 2014). Together these studies emphasise that although CBT for childhood anxiety disorders is beneficial, there is a clear need for improvement. The critical ingredient in CBT for the treatment of anxiety disorders in children and young people appears to be behavioural exposure (Kendall et al. 2005; Peris et al. 2015; Peterman et al. 2014), a controlled therapeutic technique that involves the person facing an anxiety-provoking stimulus or situation (Marks 1973). The theoretical foundations and proposed mechanisms of change that guide behavioural exposure have important implications for how the exposure session is planned, conducted and appraised (Abramowitz 2013).

Behavioural exposure was first derived from principles of associative learning through fear conditioning (learning to predict an aversive event by pairing a neutral stimulus with an aversive stimulus) and extinction (the gradual decrease in response to a conditioned fear when the stimulus is presented in the absence of the reinforcement) whereby within- and between-session fear reduction and habituation reflects successful learning (e.g. Emotion Processing Theory; Foa and McNally 1996). However, recent studies with adults have failed to find a significant relationship between within-exposure habituation and level of fear on behavioural avoidance tests at follow-up weeks to months later (Kircanski et al. 2012b). Furthermore, research with animals has suggested that, rather than facilitate successful exposure, rapid habituation may actually impede long-term learning (Woods and Bouton 2008). As such, more contemporary accounts of exposure suggest that associations learned during threat-conditioning (i.e. when the fear is acquired) are not weakened or forgotten but compete with new non-threatening associations. For example, Inhibitory Learning Theory (Craske et al. 2008) proposes that the failure to benefit from exposure is due to deficits in cognitive mechanisms, including inhibitory learning (i.e. learning which inhibits previous learning) (Craske et al. 2008), and the focus of inhibitory learning-guided exposure should be to develop new (non-threatening) associations that overshadow the excitatory (threatening) association. It is therefore proposed that the ‘success’ of exposure is reflected by effective consolidation, retrievability and generalisability of new inhibitory learning assessed during follow-up, rather than the degree of fear reduction that occurs between and within exposure sessions (Craske et al. 2014).

Inhibitory Learning Theory has highlighted several strategies that enhance extinction learning and may also increase the effectiveness of exposure within treatment. Examples include violating expectancies about harm, occasional reinforced extinction, reducing safety seeking behaviours (Salkovskis et al. 2007; Sloan and Telch 2002), stimulus variability (Culver et al. 2012; Kircanski et al. 2012b) and affect labelling (Kircanski et al. 2012a; Niles et al. 2015). Furthermore, exposure may be optimised by pharmacological strategies to enhance memory consolidation. For example, D-Cycloserine (DCS) has been shown to have a small augmentation effect on exposure-based psychotherapy for adults with anxiety, obsessive compulsive and post-traumatic stress disorders (Mataix-Cols et al. 2017).

Inhibitory Learning Theory and its application to optimise exposure has predominantly been based on research with adults and its application to younger people remains unclear. Indeed, most empirically tested CBT treatment protocols for childhood anxiety disorders apply traditional habituation-based models of exposure (e.g. Kendall et al. 2002; Kendall and Hedtke 2006). However, there are indications from animal research that different biological pathways may underpin fear extinction in children, adolescents and adults (Shechner et al. 2014) and notably, among rats, there appears to be a unique developmental period whereby fear expression and extinction are temporarily impaired during adolescence (Ganella and Kim 2014). Consistent with the animal work, recent findings from a threat-conditioning study with humans found that relative to children and adults, adolescents exhibited impairments during extinction where they were less likely to retain new, non-fearful, inhibitory information (Waters et al. 2017). The maturation of brain structures and neurotransmitter systems are likely to be responsible for these developmental distinctions in extinction as the human brain undergoes rapid developmental changes (Sowell et al. 1999). In addition to potential biological mechanisms underlying developmental differences in the process of fear extinction, there may be other factors that have an impact on the efficacy of exposure in children and young people. For example, parent or carer responses may influence the extent to which children are able to undertake the active parts of treatment by interacting with fearful situations or stimuli, through the degree to which they model and reinforce ‘brave’ approach-related behaviours and facilitate exposure in multiple contexts between sessions (e.g. at home, in school, in the community). As such it is plausible that the effectiveness of strategies to promote exposure may differ through development and that specific consideration of how to optimise exposure among children and adolescents is required.

## Summary and Aim of Review

This systematic review will explore factors associated with differential outcomes from exposure in children and young people with anxiety symptoms/disorders, and where possible examine how associations differ across this age range, by examining:

(i) Specific exposure optimisation strategies (e.g. pharmacotherapy and parental involvement)
(ii) Specific characteristics of the process of exposure (e.g. cognitive, behavioural, and therapy level characteristics)

## Method

### Protocol and Registration

The review was conducted using the Preferred Reporting Items for Systematic Reviews and Meta-Analysis of Individual Participant Data (Moher et al. 2009). The study protocol was registered with PROSPERO (CRD42018109875) and it is accessible from www.crd.york.ac.uk/PROSPERO/display_record.php?RecordID=109874

### Search Strategy

A systematic review of the literature was conducted during November 2019 using two databases *Psych-INFO and Medline (Pub-Med).* The start time was selected based on the earliest material published in the databases. The search used key exposure-based treatment terms: *exposure* in conjunction with *therapy, treatment, intervention* and behavio*, anxiety-related terms: anxi*, worry, fear*, obsess*, compul*, OCD, panic, GAD, phobi*, mute, mutism, agora*, PTSD, post-traumatic and *(stress adj2 disorder),*^2^ and terms to identify studies which involved children and adolescents: child, children, childhood, adolescen*, youth and teen*. The search results were collated in Endnote where duplicates between databases were removed.

### Inclusion Criteria

Studies were included in the review if they met the following criteria via a hierarchical coding system:

1. Written in English
2. Peer-reviewed empirical study (case studies not included)
3. Involved human participants aged between 3 and 21 years, with a mean age of ≥ 5 and ≤ 18 years
4. Focussed on typically developing children/adolescents
5. Included at least one condition with a core exposure-based intervention/treatment component (≥ 50% sessions contained exposure) that targets pre-existing fear / anxiety
6. Used at least one anxiety or fear-related outcome measure
7. Reported the statistical association between an exposure strategy or characteristic and treatment outcomes:
8. Compared exposure with and without a particular strategy (“exposure plus” condition). To ensure that observed effects were carried by features of the exposure component of an intervention/treatment (rather than other elements delivered as part of a wider treatment package, for example, psychoeducation), a strategy was defined as an “exposure strategy” if the intervention/treatment was ≥ 80% exposure OR if the strategy was administered during the exposure component of treatment (e.g. directly before, during or directly after an exposure session). For example, a study would be included if a strategy (e.g. pharmacotherapy) was introduced during the exposure component of a treatment that was 50% exposure (e.g. Leyfer et al. 2019) but excluded if the strategy was introduced during other, non-exposure components of treatment (e.g. Compton et al. 2010).
9. Reported an association between features of exposure practice (i.e. specific characteristics of exposure which have not been experimentally manipulated for example; use of safety seeking behaviour during exposure) and treatment outcome[s]. Exposure practice can also include between-session exposure-related activities (e.g. parental training to support between-session exposure)

### Study Selection

**Fig. 1:**
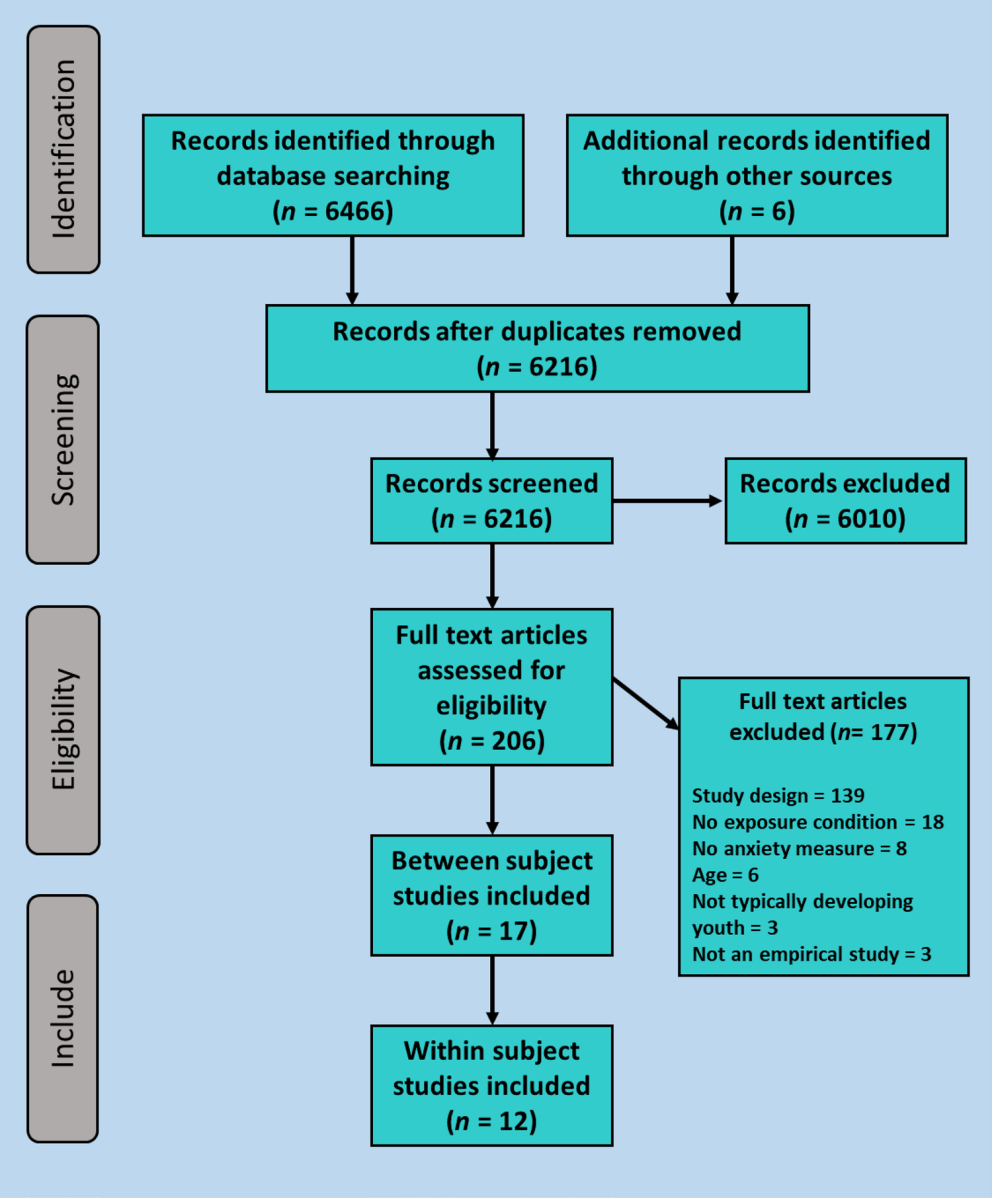
Study selection

Following a search of electronic databases, the selection process was piloted using a sample of papers. Abstracts were screened for inclusion by HP and second rated by one of three undergraduate psychology research assistants with a high level of reliability (k = 0.82). Full-text articles were screened for inclusion by HP and second rated by a postgraduate psychology research assistant with a substantial level of reliability (k = 0.73). Reference lists of the primary studies identified were reviewed to identify further potential studies of interest, and abstracts were retrieved, and full texts screened for inclusion, if appropriate. All queries regarding study eligibility were discussed and resolved between HP, CC and PW. The study selection process and the number of studies remaining at each stage is shown in Fig. 1.

### Data Extraction

For each study, the following information was extracted: study location, sample characteristics including child age and anxiety diagnostic status, intervention characteristics including treatment protocol and exposure technique, primary outcome measure(s) and where relevant, the inclusion and duration of follow-up assessment. The data was extracted by HP. Where there was missing data or additional data needed, authors of the studies were contacted.

For studies that examined the efficacy and effectiveness of exposure optimisation strategies, the following information was also extracted: (i) percentage of intervention containing exposure, and (ii) exposure PLUS (i.e. strategy) and exposure ONLY information (i.e. control). For studies that examined the relationship between exposure process variables and treatment outcomes, we also extracted exposure process variable information (e.g. safety seeking behaviour).

### Outcome Measure of Fear/Anxiety

We developed a hierarchy of preferred outcome measures:

1. Clinician ratings (CSR)—i.e. independent evaluators used a structured diagnostic interview such as the Anxiety Disorders Interview Schedule for Children – Child and Parent Versions (ADIS-C/P) (Silverman & Albano, 1996) and assigned a CSR using a 0–8 scale based on child/parent interviews.
2. Self-report measures of symptom severity/fear—i.e. child/parent questionnaires.
3. Approach/avoidance of the feared situation/stimulus—i.e. Behaviour Assessment Test (BAT).
4. Self-rating of anxiety during exposure –i.e. Subjective Units of Distress Scales (SUDS).

For each study, if multiple outcomes were reported the disorder/fear specific outcome measure with the highest rank was selected for inclusion. If a study included multiple measures from one category, the most frequently used measure across all studies in the review was selected. If a study included a self-report measure and a BAT, both measures were reported. Where child and parent measures were provided separately, both informants’ reports were included.

### Data Synthesis

Due to the heterogeneity of studies included within the review (e.g. participants, anxiety disorder/symptoms, primary outcome measures, study design, exposure strategy and/or process variable) the findings were evaluated through a narrative approach. Where possible, effect sizes were extracted or calculated for each individual study. For within-subject studies, effect sizes were reported as Pearson’s product-moment correlation coefficient (*r*). Where studies reported only standardised multiple regression coefficients, rather than correlation coefficients, we used Peterson and Brown’s (2005) imputation approach to convert B coefficients to corresponding coefficients (*r*). For experimental studies involving group comparisons, effect sizes were reported as Cohen’s *d*. Where studies did not report Cohen’s *d*, this was calculated using the available data (Cohen 1988) for each available timepoint. Where no effect size is reported it is because sufficient data were unavailable.

### Study Quality Ratings

All included studies were evaluated for methodological quality using an adapted version of Moncrieff et al. (2001)’s study quality assessment instrument. As this review included experimental and within-subject studies, adaptations were made to the instrument to account for the differences in designs. For within-subject studies, the following standards were omitted from the assessment: *Item (5) Method of Allocation; Item (6) Concealment of Randomisation; Item (8) Blinding of Subjects* and *Item (17) Information on Comparability and Adjustment for Differences in Analysis*. One item was adapted to reflect the single condition nature of these types of studies: *Item (15) Record of Number and Reason for Withdrawal (omitted ‘by group’).* For experimental studies, all items from the original instrument were included. Items 8 (blinding of subjects) and 13 (blinding of assessor) were combined. All included studies were rated by the first author (HP) and second rated by the third author (KG). Inter-rater reliability for study quality ratings was excellent (*k* = 0.98). All discrepancies and queries regarding study ratings were discussed and resolved between the authors.

## Results

### Description of Included Studies

Twenty-nine studies were identified, published between 1993 and 2019, details of which can be found in Tables 1 and 2. Seventeen (59%) of the studies used an experimental, between-subject design and 12 (41%) used a within-subject design.

**Table 1:** Study characteristics; between-subject studies

Additional component, Study authors	Exposure plus (n)	Exposure Only (n)	Age (mean)	Anxiety type	Exposure technique (Format)	Treatment approach	% sessions containing exposure^1^	Measure	Follow-up	Location	Study quality (%)
Pharmacotherapy											
*D-Cycloserine*											
Rapee et al. (2016)	EXP + DCS (*n* = 27)	EXP + PBO (*n* = 24)	7–14 (9.22)	Mixed	In vivo (Individual)	Graduated, in vivo exposure to hierarchy	100	Fear-relevant SCAS-C & SCAS-P	No	AU	54
Farrell et al. (2013)	EXP-RP + DCS (n = 9)	EXP-RP + PBO (*n* = 8)	8–18 (13.11)	OCD	In vivo (Individual)	March and Mulle (1998)	56	CY-BOCS-C-Total CY-BOCS-P-SR	1 & 3 months	AU	67
Mataix-Cols et al. (2014)	ERP + DCS (*n* = 13)	ERP + PBO (*n* = 14)	12–18 (14.96)	OCD	In vivo (Individual)	CBT for OCD	70	CY-BOCS-C/P-Total	3, 6 & 12 months	UK	80
Storch et al. (2010)	EXP-RP + DCS (*n* = 15)	EXP-RP + PBO (n = 15)	8–17 (12.2)	OCD	In vivo (Individual)	March & Mulle (1998)	60	CY-BOCS-Total^▪^ ADIS-IV-P-CSR	No	USA	54
Storch et al. (2016)	ERP + DCS (*n* = 70)	ERP + PBO (*n* = 72)	7–17 (12.8)	OCD	In vivo (Individual)	March & Mulle (1998)	70	CY-BOCS-C/P-Total	No	US	76
Leyfer et al (2019)	CBT + DCS (*n* = 14)	CBT + PBO (*n* = 10)	12–17 (14.5)	Panic	Interoceptive / in vivo (Individual)	Angelosante, Pincus, Whitton et al (2009)	50	ADIS-IV-C/P-PDA-CSR	3 months	USA	64
Scheeringa et al. (2014)	CBT + DCS (*n* = 29)	CBT + PBO (*n* = 28)	7–18 (12.5)	PTSD	Trauma-focussed (Individual)	CBT protocol	58	CPSS-C/P	3 months	USA	83
Byrne et al. (2015)	EXP + DCS (*n* = 18)	EXP + PBO (*n* = 17)	6–14 (8.08)	Specific Phobia	In vivo (Individual)	Öst, (1989)	100	BAT (steps)	1 week	AU	60
Farrell et al. (2018)	OST + DCS (*n* = 17)	OST + PBO (*n* = 18)	7–14 (10.43)	Specific Phobia	In vivo (Individual)	Öst (1989) (modified)	100	ADIS-IV-C/P-CSR	1 & 3 months	AU	74
*Sertraline*											
The POTs Study (POTS, 2004)	EXP-RP + SSRI (*n* = 28)	EXP-RP Alone (*n* = 28)	7–17 (-)^2^	OCD	In vivo (Individual)	March and Mulle (1998)	79	CY-BOCS-Total^▪^	No	USA	78
Storch et al (2013)	EXP-RP + SSRI RegSert (*n* = 14) EXP-RP SSRI SlowSert (*n* = 17)	EXP-RP PBO (*n* = 16)	7–17 (11.89)	OCD	In vivo (Individual)	March and Mulle (1998)	79	CY-BOCS-Total^▪^	No	USA	74
Observational learning											
Menzies et al. (1993)^3^	EXP + Vicarious Learning / Live Modelling (n = 12)	EXP Alone (*n* = 12)	3–8 (5.5)	Water Phobia	In vivo (Group)	Swimming lessons	100	Behaviour Rating Scale	12 weeks	AU	35
Weiss et al. (1998)	EXP + Peer mastery (*n* = 5) EXP + Peer coping (n = 5)	EXP Alone (*n* = 7)	5–7 (6.2)	Water Phobia	In vivo (Group)	Swimming lessons	100	Fear of swimming^4^	4 days	USA	33
Parental involvement											
Ollendick et al (2015)	Parent Augmented OST (*n* = 46)	OST Alone (*n* = 51)	6–15 (8.85)	Specific phobias (various)	In vivo (Individual)	Öst (1989)	100	ADIS-IV-C/P-CSR	1 & 6 months	USA	80
Ost et al. (2001)	EXP + parental involvement (*n* = 30)	EXP alone (*n* = 30)	7–17 (11.7)	Specific phobias (various)	In vivo (Individual)	Öst (1989)	100	FSSC-R–C BAT (steps)	1 Year	SE	55
Attention training to positive stimuli											
Waters et al. (2014)	OST + AT (n = 19)	OST + ATC (n = 18)	6–17 (-)^5^	Specific Phobia	In vivo (Individual)	Öst (1997)	100	ADIS-IV-C/P-Phobia-CSR	3 months	AU	71
Social skills training											
Olivares-Olivares et al. (2019)	IAFS	IAFS-R	14–17 (15.4)	Social Anxiety	In vivo, simulated & imaginal (Group)	Olivares (2005)	80	NSSFA-C	6 months	ES	62

*AT* Attention Training to positive stimuli, *ACT* Attention Training Control, *CBT* Cognitive Behavioural Therapy, *DCS* D-Cycloserine, *EXP* Exposure treatment/intervention, *EXP-RP* Exposure Response Prevention, *IAFS* intervention in adolescents with social phobia, *IAFS-R* IAFS without social skills training, *OST* One Session Treatment for Specific Phobia, *PBO* Placebo, *RegSertSSRI* sertraline at standard dosing, *SlowSertSSRI* sertraline titrated slowly, *TF-EXP* Trauma-Focussed Exposure; Anxiety Type: *OCD* Obsessive Compulsive Disorder, *PTSD* Post-Traumatic Stress Disorder; Measure: *ADIS* Anxiety Disorders Interview Schedule, *BAT* Behavioural Approach Test, *CPSS* Child PTSD Symptom Scale, *CY-BOCS* Children’s Yale-Brown Obsessive Compulsive Scale, FSSC-R = The Fear Survey Schedule for Children-Revised, *NSSFA* The number of social situations feared and/or avoided quantified based on social phobia section of the ADIS-IV, *-PDA* Panic Disorder with Agoraphobia, *SCAS* Spence Children’s Anxiety Scale, -*C* child/young person report, -*CSR* Clinician Severity Rating, -*P* parent report, *SR* self-report, c/p = child/young person and/or parent report together; ▪ = reporter not specified; Location: *AU* Australia, ES = Spain, SE = Sweden, USA = United States of America; 1 = Excluding pre-treatment, post-treatment and follow-up assessment sessions, 2 = Total sample mean age not reported,, 3 = Children either received in vivo plus vicarious exposure, vicarious exposure alone, in vivo exposure alone or assessment only control. For the purpose of the review, only findings from the in vivo plus vicarious exposure and the in vivo exposure alone conditions are reported, 4 = two instructors jointly rated each child on their fear of swimming using a scale in which scores ranged from 1 (not afraid at all) to 11 (afraid a lot), 5 = mean age not reported

**Table 2:** Study characteristics; within-subject studies

Study authors	Sample size (n)	Age (mean)	Anxiety type	Exposure technique (Format)	Treatment protocol	% Sessions containing exposure^1^	Measure	Follow-up	Location	Study quality (%)
Hedtke et al. (2009)	87	7–13 (10.32)	Generalised, Social and/or Separation	In vivo & imaginal (Individual)	Kendall (2002)	50	ADIS-C/P-CSR	No	USA	65
Peterman, Carper & Kendall (2016)	72	7–14 (10.5)	Generalised, Separation and/or Social	In vivo & imaginal (Individual)	Kendall (2002)	50	ADIS-C/P-CSR	1 year	USA	53
Peris et al. (2017)	279^2^	7–17 (10.8)	Mixed	In vivo & imaginal (Individual)	Kendall (2002)	58	PARS-C/P	No	USA	58
Tiwari et al (2013)	61	7–13 (10.5)	Mixed	In vivo & imaginal (Individual)	Kendall (2002)	50	ADIS-C/P-CSR	No	USA	55
Waters et al (2015)	26	7–12 (10)	Mixed	In vivo (Group)	Waters, Ford, Wharton, & Cobham, (2009)	50	ADIS-C/P-CSR	No	AU	55
Benito et al (2018)	111	7–17 (10.17)	OCD	In vivo (Individual)	Freeman & Garcia, (2009); March & Mulle (1998)	67–79	CY-BOCS-Total^▪^	No	USA	53
Benito et al. (2012)	18	4–8 (6.74)	OCD	In vivo (Individual)	Freeman & Garcia (2009)	67	CY-BOCS-Total^▪^	3 months	USA	53
Kircanski & Peris (2015)	35	8–17 (12.86)	OCD	In vivo (Individual)	Piacentini, Langley, & Roblek (2007)	83	CY-BOCS-Total^▪^	3 months	USA	43
Kircanski, Wu & Piacentini (2014)	40	8–17 (11.9)	OCD	In vivo (Individual)	Piacentini et al (2007)	83	CY-BOCS-Total^▪^	No	USA	53
Park et al (2014)	30	8–17 (12.2)	OCD	In vivo (Individual)	March & Mulle (1998)	60	CY-BOCS-Total^▪^	No	USA	58
Hayes et al (2017)	81	7–17.9 (12.56)	PTSD^3^ symptoms	Trauma Narrative (Individual)	Cohen, Mannarino & Deblinger (2006)	33	UPID-IV-C	No	USA	70
Ready et al. (2015)	81	7–17 (12.56)	PTSD symptoms^3^	Trauma Narrative (Individual)	Cohen, Mannarino and Deblinger (2006)	33	UPID-IV-C	9 months 12 months	USA	61

Anxiety Type: *GAD* generalised anxiety disorder, *OCD* obsessive compulsive disorder, *PTSD* post-traumatic stress disorder, *SAD* separation anxiety disorder, *SP* social phobia; Measure: *ADIS* Anxiety Disorders Interview Schedule, *CY-BOCS* Children’s Yale-Brown Obsessive Compulsive Scale, *PARS* Paediatric Anxiety Rating Scale, *UPID* The UCLA PTSD Reaction Index for DSM; -*C* child/young person report, -*CSR* Clinician Severity Rating, -*P* parent report, *SR* self-report, c/p = child/young person and parent report together; Location: *AU* Australia, *USA* United States of America, ▪ = reporter not specified, 1 = excluding pre-treatment, post-treatment and follow-up assessment sessions, 2 = *N* varied according to construct of interest: % of session with exposure *n* = 273, % of session with difficult exposure *n* = 254, cumulative dose of exposure *n* = 241, child compliance *n* = 254, child mastery *n* = 254, 3 = PTSD symptoms = not diagnosis. Included participants scored ≥ 17 on the UPID-A or endorsed 3/9 PTSD symptoms based on an independently verified (e.g. through child welfare) trauma

Sixteen studies (55%) included participants with a broad age range (e.g. 7–17 years), 6 (21%) included children and early adolescents (e.g. 6–14 years), 4 (14%) only included young children (e.g. 3–8 years) and 3 (10%) only included adolescents (e.g. aged 12–17 years).

Twenty-five (86%) studies included clinical samples that met diagnostic criteria for anxiety disorders, and all of these studies delivered treatment using a manualised protocol (e.g. Angelosante et al. 2009; Kendall and Hedtke 2006; March and Mulle 1998; Öst 1989, 1997). Four (14%) of the studies did not conduct diagnostic assessments; two used an exposure-based intervention (e.g. gradual in vivo exposure) and two used a manualised trauma-focussed treatment protocol (i.e. Cohen et al. 2006). Twenty-two (76%) studies used an intervention/treatment protocol that included exposure in more than 50% of the sessions. However, only three (10%) studies reported the association between the amount of time spent on exposure/the number of exposures and treatment outcomes. Twenty-one (72%) studies used an intervention/treatment protocol that involved parents, 5 (17%) only included children/adolescents and 3 (10%) did not report parent involvement.

Twenty-eight (97%) of the studies included a post-exposure assessment, and 16 (55%) included one or more follow-up assessments ranging from 4 days to 1-year post-exposure.

### Quality Ratings

As shown in Tables 1 and 2, quality ratings ranged widely from 33 to 83%. Particular areas of weakness in study quality related to a lack of information on and inclusion of withdrawals, fidelity, sample size, compliance and power calculation. There was also commonly a lack of information about side effects, although these were more commonly reported in studies of pharmacological approaches. Notably, only 7 studies included a follow-up > 3 months post-treatment completion and only 2 studies included a generalisation assessment (Table 3).

**Table 3:** Differences between conditions and associations between characteristics of exposure and anxiety outcomes, by assessment timepoint

Construct	Study	Anxiety Type	Studies *(n)*	Effect Size by assessment timepoint		Cohen’s *d* or r
Before exposure						
DCS 1 h *before* exposure	Farrell et al. (2013)	OCD (difficult to treat)	1	PT = 0.00 1 m = -0.50 3 m = -0.40	NE Medium Small	*d*
				*Pa:* PT = − 0.15 *Pa:* 1 m = − 0.69^□^ *Pa:* 3 m = 0.22	NE Large Small	*d*
	Storch et al (2010)	OCD	2	PT = -0.67	Medium	*d*
	Storch et al (2016)			PT = ⋄	-	*-*
	Leyfer et al (2019)	Panic	1	PT = 0.18^□^ 3 m = 0.04^□^	NE NE	*d*
	Scheeringa and Weems (2014)	PTSD	1	PT = 0.71^□^ 3 m = 0.62^□^	Medium Medium	*d*
	Byrne et al. (2015)					
	*Same context*	Specific Phobia	1	1w = 0.19	Small	r
	*Novel context*			1w = -0.37*	Medium	r
DCS at the *commencement* of exposure	Farrell et al (2018)	Specific Phobia	1	PT = ⋄ 1 m = ⋄ 3 m = ⋄	- - -	*-* *-* *-*
	Rapee et al (2016)	Mixed	1	PT = 0.56^□^	Medium	*d*
				*Pa:* PT = -0.23^□^	Small	
Sertraline	Storch et al (2013) *RegSert*	OCD	2	PT = −0.02^□^	NE	*d*
	*SlowSert*			PT = 0.23^□^	Small	*d*
	The POTS Study (2004)			PT = −0.31^□^**	Small	*d*
Attention Training to Positive Stimuli (ATP)	Waters et al (2014)	Specific phobia	1	PT = 0.25^□^ 3 m = 0.12^□^	Small NE	*d*
Observational Learning	Menzies and Clarke (1993) *Same context*	Fear of Water	2	PT = ⋄ 12w = ⋄*	-	-
	*Novel Context*			1w = ⋄	-	
	Weiss et al (1998) *Peer Mastery*			PT = -0.60^□^* 4d = -0.42^□^	Medium Small	*d*
	*Peer Coping*			PT = -0.50^□^* 4d = -0.11^□^	Medium NE	*d*
Social Skills Training	Olivares-Olivares et al (2019)	Social	1	PT = 1.06Δ*** 6 m = 1.00Δ*** 12 m = 0.95Δ***	Large Large Large	*d*
Preparation	Tiwari et al (2013)	Mixed	1	PT = 0.15Δ♦	Small	r
Within exposure						
Quantity of Exposure	Hedtke et al (2009)	Mixed	1	PT = ⋄*	–	–
	Benito et al (2018)	OCD	2	PT = ⋄	–	–
	Kircanski & Peris (2015)			PT = ⋄ 3 m = ⋄	– –	–
Time spent on Exposure	Hedtke et al (2009) *Average length of exposure tasks per session*	Mixed	1	PT = ⋄	–	–
	Benito et al (2018) *Duration of exposures*	OCD	2	PT = ⋄	–	–
	Kircanski & Peris (2015) *Minutes spent on ERP tasks per session*	OCD		PT = ⋄ 3 m = ⋄	– –	–
Cumulative Dose of Exposure	Peris et al (2017)	Mixed	1	PT = ⋄***	-	-
Percentage of Session with Exposure	Peris et al (2017)	Mixed	1	PT = ⋄***	-	-
Percentage of Session with Difficult Exposure	Peris et al (2017)	Mixed	1	PT = ⋄***	-	-
Proportion of Session with Combined Exposure	Kircanski & Peris (2015)	OCD	1	PT = ⋄ 3 m = ⋄	- -	-
Frequency of Exposure Task Type	Hedtke et al (2009)	Mixed	1	PT = ⋄	-	-
Location of Exposure Task	Hedtke et al (2009)	Mixed	1	PT = ⋄	-	-
Safety Seeking	Hedtke et al (2009)	Mixed	1	PT = -0.37Δ♦*	Medium	r
Cognitive Strategy	Benito et al (2012)	OCD	1	PT = ⋄	-	-
Avoidance Statement			1	PT = ⋄	-	-
Avoidant Behaviour			1	PT = ⋄	-	-
Compliance	Peris et al (2017)	Mixed	1	PT = ⋄***	-	-
Mastery	Peris et al (2017)	Mixed	1	PT = ⋄***	-	-
Coping	Hedtke et al (2009)	Mixed	1	PT = 0.11Δ♦	Small	r
Processing	Hayes et al (2017)	PTSD	2			
	Negative Emotion			PT = 0.21♦	Small	r
	Avoidance			PT = 0.00♦	NE	r
	Ruminative Processing			PT = 0.06♦	NE	r
	Decentring			PT = -0.02♦	NE	r
	Ready et al (2015)					
	Overgeneralisation (beliefs)			PT = 0.15 6 m = 0.24* 9 m = -0.12 1y = 0.08	Small Small Small NE	r
	Accommodation			PT = -0.12 6 m = -0.16 9 m = 0.09 1y = 0.19	Small Small NE Small	r
Initial distress	Kircanski & Peris (2015)	OCD	1	PT = ⋄ 3 m = ⋄	- -	-
Fear activation	Hedtke et al (2009)	Mixed	2	PT = ⋄	-	-
	Peterman et al (2016)			PT = -0.11 1y = -0.16	Small Small	r
	Benito et al (2018)	OCD	1	PT = ⋄	-	-
Fear Reduction	Peterman et al (2016)	Mixed	2			
	*Between session*			PT = 0.00 1y = -0.05	NE NE	r
	*Within session*			PT = -0.20 1y = -0.17	Small Small	r
	Waters et al (2015)			PT = 0.42Δ*	Medium	r
	Benito et al (2018)	OCD	2	PT = ⋄*	–	-
	Kircanski & Peris (2015)			PT = ⋄ 3 m = ⋄	– –	–
50% Rule	Peterman et al (2016)	Mixed	1	PT = -0.08 1y = -0.05	NE NE	r
Variability of distress	Waters et al (2015)	Mixed	1	PT = 0.50Δ**	Large	r
	Benito et al (2018)	OCD	2	PT = ⋄	-	-
	Kircanski and Peris (2015)			PT = ⋄ 3 m = -0.40Δ♦*	- Medium	- r
Expected minus actual distress	Kircanski & Peris (2015)	OCD	1	PT = ⋄ 3 m = ⋄	- -	-
Final distress	Kircanski & Peris (2015)	OCD	1	PT = ⋄ 3 m = ⋄	- -	-
Parent involvement	Hedtke et al (2009)	Mixed	1	PT = ⋄	-	-
	Benito et al (2012) *Discourage avoidance*	OCD	1	PT = ⋄ 3 m = 0.84Δ**	**-** Very Large	**-** r
	*Externalising statements*			PT = ⋄ 3 m = ⋄	- -	-
	Ollendick et al (2015)	Specific Phobia	2	PT = 0.24 1 m = 0.17 6 m = 0.20	Small NE Small	*d*
	Ost et al. (2001)			PT = 0.25^□^ 1y = 0.17^□^ _*BAT*_ PT = ⋄ _*BAT*_ 1y = ⋄	Small NE - -	*d* *-*
Therapist Involvement	Benito et al (2012) *Discourage avoidance*	OCD	1	PT = ⋄ 3 m = 0.73Δ**	**-** Very Large	r
	*Unrelated Talk*			PT = ⋄ 3 m = ⋄	- –	-
	*Exposure comments (to increase anxiety)*			PT = ⋄ 3 m = ⋄	- -	-
After exposure						
Child processing	Tiwari et al (2013)	Mixed	1	PT = 0.18Δ♦*	Small	r
DCS after exposure	Mataix-Cols et al. (2014)	OCD	1	PT = 0.07 3 m = 0.10 6 m = 0.19 1y = 0.15	NE NE NE NE	*d*
Between sessions						
Fear Reduction	Peterman et al (2016)	Mixed	1	PT = 0.00 1y = −0.05	NE NE	r
	Kircanski & Peris (2015)	OCD	2	PT = ⋄ 3 m = ⋄	**–**	–
	Kircanski, Wu and Piacentini (2014)			PT = ⋄**	–	–
				*Pa*PT = ⋄***	–	–
Homework Compliance	Park et al (2014)	OCD	1	PT = -0.65	Large	r

*Pa* = parent report; PT = post-treatment; Follow-up: d = day, w = week, m = month, y = year; For between-subject studies (*d*): negative effect indicates lower anxiety level for “EXP plus” condition; positive effect indicates lower anxiety level for “EXP only/placebo” control condition; Δ = measure reported as change between assessment timepoints so that a higher score indicates a greater reduction in anxiety; ♦ = r imputed from β coefficients using Peterson and Brown’s (2005) imputation approach; □ = effect size calculated using available data; ⋄ = insufficient data available to calculate effect size; *p < .05, ** p < .01, *** *p* < .001; NE = did not meet the threshold for a small effect, *BAT* Behavioural Approach Test

### Exposure Characteristics and Optimisation Strategies

#### Before Exposure

Fourteen studies examined associations between pre-exposure variables (administration of pharmacotherapy, modelling, attention training, social skills training and exposure preparation) and exposure outcomes.

### Pharmacotherapy

#### D-Cycloserine

Six studies examined the effect of DCS administration *one hour prior* to commencing exposure (Byrne et al. 2015; Farrell et al. 2013; Leyfer et al. 2019; Scheeringa and Weems 2014; Storch et al. 2010, 2016), and two studies examined the effect of DCS administration *at the beginning* of each exposure session (Farrell et al. 2018; Rapee et al. 2016) All eight studies used a double blind, placebo-controlled design.

One study found a significant facilitative effect of DCS administered *1 hour before* exposure on treatment outcomes (Byrne et al. 2015). In the treatment of specific phobias, Byrne et al. (2015) found that children and young adolescents (aged 6–14 years) who received DCS administration prior to prolonged exposure did not perform significantly better than those who received a placebo control during a behaviour approach test (BAT) at 1-week follow-up, with a small effect size. However, when the stimulus was presented in a novel context (i.e. a different stimulus outdoors with no parent present) children who received DCS performed significantly better than those in the control group during the BAT, with a medium effect size. However, in a study which included children and adolescents (aged 8–18 years) with ‘difficult to treat’ OCD, Farrell et al. (2013) did not find a significant facilitative effect of DCS on OCD severity immediately post-treatment (did not meet a threshold for a small effect) nor at 1- and 3-month follow-up (medium and small effect size respectively). A large (though non-significant) facilitative effect of DCS was observed at 1-month follow-up, based on a parent report measure. In a third study, Storch et al. (2010) found that children and adolescents (aged 8–17 years) who received DCS before each of 7 sessions of exposure did not have significantly greater improvements in OCD severity immediately post-treatment compared to those in a placebo condition, although the pattern of results suggested an advantage for DCS with a medium effect size. No significant differences were observed in the rate of improvement over time and no follow-up assessment was conducted. In a further study, Storch et al. (2016), found no significant difference in OCD severity for children and adolescents (aged 7–17 years) between those who received DCS and placebo control at post-treatment. Again, no follow-up assessment was conducted.

One study focussed on the treatment of panic disorder in adolescents (aged 12–17 years) (Leyfer et al. 2019) and found no evidence for a significant facilitative effect of DCS on disorder severity at post-treatment, nor at 3-month follow-up, compared to placebo control, with neither meeting the threshold for even a small effect.

One study focussed on the treatment of PTSD in children and adolescents (aged 7–18 years) (Scheeringa and Weems 2014) and found no evidence of a significant facilitative effect of DCS taken one hour prior to narrative exposure, on PTSD symptoms at post-treatment or 3-month follow-up. However, the pattern of results indicated that children with high PTSD symptom scores who received the placebo, had lower symptoms both post-treatment and at 3-month follow-up compared to those who received DCS, with a medium effect size.

Two studies involving children and young adolescents (7–14 years), with mixed anxiety disorders (Rapee et al. 2016) and specific phobias (Farrell et al. 2018), examined the effect of DCS administered *at the beginning* of exposure. Findings from both studies suggested that DCS administration at the beginning of exposure does not significantly enhance outcomes post-treatment. Specifically, in the treatment of broad anxiety disorders, Rapee et al. (2016) found that those who received a placebo had (non-significantly) lower anxiety symptoms (based on child report) at post-treatment than those who received DCS, with a medium effect. However, the opposite pattern was found with a parent report measure, in which those who received DCS had (non-significantly) lower anxiety symptoms at post-treatment, with a small effect. In the treatment of specific phobias (Farrell et al. 2018), there were no significant benefits of DCS compared to placebo control in anxiety severity across any timepoints. However, when age was examined as a moderator, significant differences were found between treatment conditions whereby improvements at post-treatment among the placebo group appeared to be accounted for by positive effects among adolescents, whereas improvements at the 1-month follow-up among the DCS group appeared to be accounted for by positive effects among pre-adolescent children.

#### Sertraline

Two studies examined the post-treatment effect of sertraline augmented exposure-based CBT for children and adolescents aged 7–17 years with OCD (Storch et al. 2013; The Pediatric OCD Treatment Study (POTS) 2004). While POTS (2004) found a significant, facilitative effect of sertraline augmented treatment compared to CBT alone, with a small effect size, the findings were not replicated by Storch et al. (2013) who did not find a significant difference in OCD severity between those who received sertraline (regular or slow dosing) and placebo control. The pattern of results indicated that children and adolescents who received the placebo, had lower OCD severity at post-treatment compared to those who received slow sertraline dosing, with a small effect, but marginally greater OCD severity compared to those who received regular sertraline dosing, although neither effect was significant.

#### Attention Training to Positive Stimuli

One study compared the effects of a pre-treatment session in which children and adolescents (aged 6–17 years) received attention training to positive stimuli (i.e. modifying attention biases away from threatening, towards neutral, facial stimuli) or an attention training control, prior to a single session of exposure on outcomes for a specific phobia (Waters et al. 2014). There were no significant between group differences at post-treatment nor 3-month follow-up (with only the post-treatment results meeting the threshold for a small effect); however a greater post-treatment bias towards positive stimuli significantly predicted lower phobia severity at 3-month follow-up for children who received the pre-treatment attention training, with a small effect size.

#### Observational Learning

Two studies examined whether observational learning enhances exposure for children fearful of swimming (Menzies and Clarke 1993; Weiss et al. 1998). Weiss et al. (1998) allocated children (aged 5–7 years) to either in vivo exposure plus peer mastery modelling (PMM), peer coping modelling (PCM), or exposure alone (IVE). Children in the peer modelling groups either watched a video of a peer engage in highly competent (PMM) or less competent (PCM) swimming-related behaviour. Both mastery and coping modelling significantly enhanced exposure immediately post-exposure, both with a medium effect, but this was no longer significant by the 4-day follow-up, and effect sizes at this timepoint were small. A second study with 3 to 8-year-old children found evidence that modelling may enhance in vivo exposure long term (Menzies and Clarke 1993). Children received either vicarious exposure (i.e. observed an adult swimming instructor model display competent, fearless behaviour while in a swimming pool) (IVVE) or a non-related task (i.e. observed a variety of card games) (IVE) prior to 15 min of gradual in vivo exposure. There were no significant between group differences in fearful behaviour post-intervention or the extent to which treatment gains generalised to a novel swimming pool scenario; however, maintenance of fear reduction (i.e. approach-related behaviour) was significantly poorer from post- to 12-week follow-up in the IVE condition compared to the IVVE condition.

#### Social Skills Training

One study compared the effects of a pre-exposure social skills training by allocating adolescents (aged 14–17 years) with social anxiety disorder to receive either exposure-based CBT plus social skills training (e.g. starting/maintaining conversations, assertiveness, paying and accepting compliments, making and keeping friends and training in public speaking), or exposure-based CBT alone (Olivares-Olivares et al. 2019). Compared to the CBT alone group, the CBT plus social skills training group had significantly greater improvements in the number of social situations feared and/or avoided at post-treatment, 6- and 12-month follow-up, all with large effect sizes.

#### Exposure Preparation

In the treatment of children and young adolescents (aged 7–13 years) with mixed anxiety disorders, Tiwari el al (2013) found that the amount of pre-exposure preparation (i.e. a broad, overall quality measure including activities such as an explanation of the rationale for exposure, selecting the exposure task, role-playing/practicing with the therapist and discussion and/or selection of a reward) was not significantly related to anxiety severity immediately post-treatment, with a small effect.

### Within Exposure

Eleven studies examined associations between within-exposure variables (features of exposure tasks, child factors, distress, parent and therapist involvement) and outcomes.

#### Features of Exposure Tasks

Four studies looked at associations between the characteristics of exposure tasks and treatment outcomes (Benito et al. 2018; Hedtke et al. 2009; Kircanski and Peris 2015; Peris et al. 2017).

#### Quantity of Exposure

Three studies examined the association between the amount of time spent on exposure and the number of exposures and treatment outcome for OCD (Benito et al. 2018; Kircanski and Peris 2015) and anxiety disorders (Hedtke et al. 2009). Notably, the studies differed in how the number of exposures were quantified. For example, Benito et al. (2018) measured the cumulative sum of all instances of fear change per participant, whereas Hedtke et al. (2009) measured the total number of exposure tasks per session. Nonetheless, the amount of time spent on exposure was not significantly associated with better outcomes in any study (Benito et al. 2018; Hedtke et al. 2009; Kircanski and Peris 2015). Specifically, neither Benito et al. (2018) nor Kircanski and Peris (2015) found that number of exposures was significantly associated with better OCD outcomes (for children aged 7–17 and 8–17 years respectively) at post-treatment or 3-month follow-up (in Kircanski and Peris 2015). Furthermore, Hedtke et al. (2009) found that a greater number of exposure tasks per session was significantly associated with *less* change in anxiety severity from pre to post-treatment in the treatment of children and young adolescents (aged 7–13 years) with mixed anxiety disorders.

The amount of exposure has also been quantified based on the proportion of treatment that was spent on exposure in two studies (Peris et al. 2017; Kircanski and Peris 2015). Peris et al. (2017) found that for children and adolescents (aged 7–17 years) with mixed anxiety disorders, a greater percentage of sessions with exposure (therapist rated), and a greater “cumulative dose” of in vivo exposure, were both significantly associated with improved anxiety severity post-treatment. There was also evidence that a greater proportion of sessions containing exposures that the young person categorised as ‘difficult’ was significantly associated with improved anxiety severity post-treatment (Peris et al. 2017). Kircanski and Peris (2015) focussed specifically on the proportion of combined exposures per session (i.e. single exposure tasks that target more than one symptom or stimulus simultaneously) and found no evidence that the proportion of combined exposures was significantly associated with outcomes for children and adolescents (aged 7–18 years) with OCD post-treatment or at a 3-month follow-up.

#### Exposure Task Type

One study with 7–13-year-old children with mixed anxiety disorders (Hedtke et al. 2009), investigated the way in which the exposure was delivered (i.e. imaginal vs in vivo) and degree to which the exposure task matched the principal diagnosis, and found no evidence that either were significantly associated with better outcomes post-treatment.

#### Location

There was no evidence that the location of exposure (i.e. imaginal or in vivo exposure occurring within or outside the therapy room) was significantly associated with treatment outcomes, based on one study with children and young adolescents with mixed anxiety disorders (Hedtke et al. 2009).

### Child Factors

Three studies investigated the association between child behaviour during exposure and outcomes (Benito et al. 2012; Hedtke et al. 2009; Peris et al. 2017).

#### Safety Seeking and Avoidance

Two studies used video-taped exposure sessions to examine the relationship between child behaviours during exposure and outcomes. Hedtke et al. (2009) found that safety seeking behaviour (e.g. checking for exits or bathrooms, carrying safety aids, hand sanitizer or repeatedly seeking verbal reassurance from others) during exposure was significantly associated with *less* change in anxiety severity from pre to post-treatment, with a medium effect size. No follow-up assessments were conducted. In a second study, Benito et al. (2012) found that child avoidance statements (i.e. child statement indicating avoidance or distraction from exposure stimulus such as, “Is this going to hurt me?” and “Can I use the bathroom?”) and avoidance behaviours (e.g. avoiding contact with the exposure stimulus or using compulsive behaviour) were not significantly associated with OCD severity at post-treatment or 3-month follow-up.

#### Cognitive Strategy

One study investigated children’s use of cognitive strategies to try to lower anxiety (e.g. “I know I won’t actually hurt anyone because I’ve never done it before”) during exposure and found no significant association with OCD severity post-treatment or at 3-month follow-up (Benito et al. 2012).

#### Compliance, Mastery and Coping

Peris et al. (2017) found that therapist ratings (7-point scale) of child compliance (i.e. how well the child completed the requirements of therapy as specified by the therapist) and mastery (i.e. how well the child mastered the information/skill presented during the session) within-exposure sessions were significantly associated with greater anxiety severity improvements at post-treatment for children and adolescents (7–17 years) with mixed anxiety disorders.

There was no evidence that child coping behaviour (i.e. behaviour that is used before or during exposure that is intended to help manage, not escape or avoid, anxiety or fear) during exposure was significantly associated with changes in anxiety severity from pre to post-treatment and the effect was small (Hedtke et al. 2009).

#### Processing

Two studies with young people (7–17 years) with PTSD looked at associations between child processing during the narrative phase of treatment (i.e. exposure) and treatment outcomes (Hayes et al. 2017; Ready et al. 2015). Ready et al. (2015) found evidence that greater overgeneralisation (i.e. global, exaggerated beliefs of self, others, or the world related to a traumatic event) during exposure was significantly associated with poorer PTSD symptom outcomes six months post-treatment, with a small effect size, but not immediately post-treatment or at 9-month or 12-month follow-up (with only post-treatment and 9-month follow-up meeting threshold for a small effect). There was no evidence that more accommodation (defined as the extent to which the individual shows a balanced view of the self, others, or the world) during exposure was significantly associated with short- or long-term outcomes (all but the 9-month follow-up met the threshold for a small effect size). Reanalysis of data from the same trial found no evidence that unproductive processing (i.e. rumination and avoidance), productive processing (i.e. decentring) or levels of negative emotion expressed during exposure significantly predicted PTSD symptom outcomes, with only negative emotion meeting threshold for a small effect (Hayes et al. 2017).

### Distress

Five studies investigated the relationship between child distress during exposure and treatment outcomes (Benito et al. 2018; Hedtke et al. 2009; Kircanski and Peris 2015; Peterman et al. 2016; Waters et al. 2015).

#### Initial Distress

Kircanski and Peris (2015), found that initial distress (i.e. the SUDS level for the first exposure response prevention (ERP) task during the first ERP session) was not significantly associated with OCD severity immediately post-treatment in children and adolescents (aged 8–17 years).

#### Fear Activation

Three studies examined the association between fear activation (i.e. the highest anxiety rating) during exposure and treatment outcomes (Benito et al. 2018; Hedtke et al. 2009; Peterman et al. 2016). None of the studies found significant associations between fear activation and OCD or mixed anxiety symptom severity post-treatment (Benito et al. 2018; Hedtke et al. 2009; Peterman et al. 2016) and, in the one study where an effect size was available, this did reach a small effect at post-treatment and at 1-year follow-up (Peterman et al. 2016). However, secondary analysis of the data in Peterman et al. (2016) did find that greater initial fear activation was significantly associated with lower anxiety severity at 1-year follow-up for children with a diagnosis of separation anxiety and/or social anxiety disorder, but not for those with generalised anxiety disorder.

#### Fear Reduction

Four studies looked at the relationship between within-exposure fear reduction and treatment outcomes (Benito et al. 2018; Kircanski and Peris 2015; Peterman et al. 2016; Waters et al. 2015). There is mixed evidence from two studies with young people with OCD that greater within-exposure fear reduction across treatment is associated with more positive outcomes. Benito et al. (2018) found that more ‘habituation’ (which they operationalised as fear reduction at a time when no explicit strategies were applied to cope with/address fear) across a number of exposure sessions (mean = 4) was associated with significantly greater reductions in OCD severity at post-treatment. There were no significant associations between peak-minus-end fear, (i.e. the end fear subtracted from the highest fear during the exposure), and the number of exposures ending with no fear across treatment and treatment outcomes. Kircanski and Peris (2015) found no significant association between the amount that distress decreased over the first three exposure tasks and OCD severity at post-treatment, nor at 3-month follow-up.

In the treatment of children and young adolescents (aged 7–12 years) with mixed anxiety disorders, Waters et al. (2015) found that more within-session habituation across exposure sessions was associated with significantly greater improvements in anxiety severity at post-treatment, with a medium effect size. However, in the treatment of children and adolescents (aged 7–14 years), Peterman et al. (2016) found a small, non-significant relationship between within-session habituation and anxiety severity at post-treatment, and 1-year follow-up.

#### 50% Rule

In the treatment of children and young adolescents with mixed anxiety disorders, Peterman et al. (2016) found that greater use of the 50% rule (i.e. SUDS ratings reduction of at least 50% before ending the exposure task) was not significantly related to anxiety severity at post-treatment or at 1-year follow-up, and this did not meet threshold for a small effect.

#### Variability of Distress

Three studies investigated emotional variability during exposure (Benito et al. 2018; Kircanski and Peris 2015; Waters et al. 2015). Notably, each study differed in terms of how emotional variability was measured; Benito et al. (2018) measured the cumulative sum of all observer rated fear increases and decreases throughout exposure, Kircanski and Peris (2015) calculated the average difference between the maximum and the minimum SUDS level for each ERP session, and Waters (2015) used the standard deviation of the four critical exposure SUDS ratings during each exposure activity, averaged across 5 exposure activities. There is evidence from two studies that greater variability of distress during exposure is associated with better outcomes (Kircanski and Peris 2015; Waters et al. 2015). At post-treatment, Waters et al. (2015) found a significant association between greater emotional variability and improved mixed anxiety severity immediately post-treatment, with a large effect size, although this association was not found by Benito et al. (2018) or Kircanski and Peris (2015). However, at 3-month follow-up, Kircanski and Peris (2015) did find that greater emotional variability in distress was significantly associated with reductions in OCD severity, with a medium effect size.

#### Differences between Expected and Actual Distress

In the treatment of children and adolescents with OCD, Kircanski and Peris (2015) found that the discrepancy between expected and actual distress, conceptualised as the average difference between anticipated distress and actual distress levels for each ERP session, was not significantly associated with improved OCD severity at immediate post-treatment, nor 3-month follow-up.

#### Final Distress

Kircanski and Peris (2015) found that final distress (i.e. the distress level for the final exposure task during the last exposure session) was not significantly associated with improved OCD severity a post-treatment, or at a 3-month follow-up.

### Therapist Involvement

One study investigated the association between therapist behaviour during exposure and OCD severity (Benito et al. 2012). The study found that post-treatment, none of the following therapist behaviours were significantly associated with outcomes: addressing accommodation, encouraging the use of cognitive strategies, unrelated talk, exposure comments, accommodation behaviour, externalising talk and discouraging avoidance. However, at the 3-month follow-up, discouraging avoidance (i.e. discouraging the child from decreased mental or actual avoidance of exposure stimulus, by statements such as “Keep looking at the sink”) was significantly associated with a greater reduction in OCD symptoms, with a very large effect size. No other significant associations were found.

### Parent Involvement

Four studies looked at the association between parent involvement during exposure and outcomes (Benito et al. 2012; Hedtke et al. 2009; Ollendick et al. 2015; Öst et al. 2001).

In the treatment of childhood phobias, two studies compared child-focussed one session exposure to parent augmented exposure (Ollendick et al. 2015; Öst et al. 2001). In the study by Öst et al. (2001) parents were present during the session to function as a supportive figure and to be directly involved (e.g. modelling) where appropriate, whereas parents in Ollendick et al. (2015) were provided with psychoeducation and taught strategies to reinforce courageous approach behaviours. Both of the studies found that children and adolescents aged 7–17 (Öst et al. 2001) and 6–15 years (Ollendick et al. 2015) who received parent augmented exposure did not significantly differ on reported fears and anxiety severity, respectively, at any timepoint from those who received child-focussed treatment alone (with small effects in favour of child only exposure at post-treatment for both studies, and at 6-month follow-up in Ollendick et al. 2015). Notably, in the study by Öst et al. (2001), the child-alone condition had significantly more clinically improved children and adolescents on a behavioural approach test (steps towards the feared animal, object or situation) at post-treatment compared to the parent augmented exposure group; however, no significant differences were found at 1-year follow-up. There is no evidence that parent presence during exposure (i.e. whether at least one parent or guardian was present during the planning or implementation of the exposure task) is associated with anxiety severity in the treatment of children and adolescents with mixed anxiety disorders (Hedtke et al. 2009). As for parent behaviours, Benito et al. (2012) found that none of 8 measured behaviours were associated with better outcomes in the short term but at 3-month follow-up, parent discouraging of avoidance was significantly associated with a greater reduction in OCD symptoms, with a very large effect size.

### Following Exposure

Five studies looked at the association between post-exposure characteristics (child processing, pharmacotherapy and fear reduction) and outcomes.

#### Child Processing

Tiwari et al. (2013) found that the child’s post-exposure processing (i.e. discussion of their experience and distress ratings) was significantly associated with greater improvements in anxiety severity from pre to post-treatment, with a small effect size.

#### Pharmacotherapy

In a double blind, placebo-controlled study of adolescents (age 12–18 years) with OCD, Mataix-Cols et al. (2014) found that DCS administration *immediately after* exposure (sessions 3–12) did not significantly facilitate short or long-term outcomes, and none of the associations met the threshold for a small effect size.

#### Fear Reduction

Three studies examined whether greater between-session reduction of fear was associated with better treatment outcomes (Kircanski and Peris 2015; Kircanski et al. 2014; Peterman et al. 2016). Results were inconsistent but notably studies differed in how between-session fear reduction was calculated. For example Kircanski et al. (2014), used the average child distress ratings for all obsessive compulsive symptoms per session, whereas Peterman et al. (2016) used the maximum child distress rating per session, to calculate differences between sessions. Kircanski et al. (2014) found a significant association between greater between-session reductions in both child and parent reported distress, and improved OCD severity outcomes at post-treatment. However, this finding was not replicated by Kircanski and Peris (2015) or Peterman et al. (2016) at post-treatment, 3-month or 1-year follow-up respectively. It is also important to note that, unlike Kircanski et al. (2015), Kircanski and Peris (2015) did not find a significant decrease in distress between sessions (e.g. as a result of exposures increasing in difficulty over the course of treatment) which may, in part, account for null findings.

### Between Session

One study looked at the association among between-exposure characteristics and outcomes.

#### Homework

In a reanalysis of data from a previous study (Storch et al. 2010), Park et al. (2014) found a significant association between more homework compliance and greater reductions in OCD severity outcomes at post-treatment, with a large effect size.

## Discussion

This review synthesised findings from 29 studies, examining factors associated with outcomes from exposure-based interventions in children and young people with anxiety symptoms/disorders. We found some preliminary evidence for specific optimisation strategies, such as dropping safety behaviours, parents and therapists discouraging avoidance, and the use of homework. However, not one significant finding was replicated by another study for the same timepoint using the same methodology. To a large degree, this lack of replication reflects a lack of consistency across studies around conceptualisations, methodological approaches, and outcome measures, making it difficult to make meaningful comparisons between studies and limiting the scope for drawing meaningful, reliable conclusions.

Much of the literature used a habituation-based model to examine exposure characteristics and, in line with animal (Woods and Bouton 2008) and adult human (Craske et al. 2008; Culver et al. 2012; Kircanski et al. 2012b) research, the studies generally failed to support a role of habituation-based fear reduction in the successful treatment of child and adolescent anxiety disorders. For example, there was no evidence of a significant association between treatment outcome and initial fear activation (Benito et al. 2018; Hedtke et al. 2009; Peterman et al. 2016) or ‘the 50% rule’ (Peterman et al. 2016), and evidence was mixed for both within-session fear reduction (Benito et al. 2018; Kircanski and Peris 2015; Peterman et al. 2016; Waters et al. 2015) and emotional variability during exposure (Benito et al. 2018; Kircanski and Peris 2015; Waters et al. 2015).

On the other hand, we found some evidence for the use of exposure strategies derived from inhibitory learning theory (e.g. Craske et al. 2008; Vervliet et al. 2013). Consistent with experimental research with adults (Salkovskis et al. 2007; Sloan and Telch 2002), reducing the young person’s use of safety behaviours and parents/therapists’ discouragement of avoidance during exposure (Benito et al. 2012; Hedtke et al. 2009) were significantly associated with enhanced outcomes. However, support for the use of pharmacological strategies to enhance memory consolidation was mixed (e.g. Byrne et al. 2015; Farrell et al. 2013; Scheeringa and Weems 2014; Storch et al. 2010, 2016). Some studies found that characteristics that appeared to be consistent with inhibitory learning strategies were associated with improved exposure outcomes, such as violation of expectancies (Tiwari et al. 2013); however, the studies were not set up using this theoretical approach or terminology and therefore it is not clear whether the findings can truly be accounted for by inhibitory learning strategies.

### Strengths and Limitations

This is the first systematic exploration of the current state of empirical literature on optimising exposure for childhood anxiety disorders. Strengths include the broad inclusion criteria (e.g. all anxiety disorders/pre-existing fears, any anxiety outcome measure) and the use of a hierarchy of pre-specified outcome measures to determine selected outcomes. Where possible, effect sizes and quality ratings were considered in the interpretation of findings, particularly where findings were mixed.

Nevertheless, the review only included up to two outcome measures for each study therefore significant effects identified using different measures may have been overlooked. There was wide variation in the quality of studies included, with lower quality studies failing to report on patient withdrawals from the study, compliance with the treatment, and therapist fidelity to treatment. In these instances, it is not possible to determine whether exposure strategies altered the amount of attrition from treatment or whether a lack of therapist fidelity or patient compliance may have washed out any potential effects. Crucially, the majority of studies did not report or adequately describe a power calculation and many studies lacked a sufficiently large sample size to detect potentially clinically meaningful effects. Another key limitation is the small number of available studies and, within those, the extensive variation in the conceptualisation of key factors. That is, although several studies looked at similar constructs (e.g. within-session reduction of fear, quantity of exposure), there was variation in how these constructs were measured. This resulted in mixed findings and limited comparability between studies. The majority of studies did not include a follow-up assessment, and of those that did; only seven (24%) included a follow-up beyond 3 months. Therefore, at this stage, inferences can only be based on short-term outcomes. Only one study examined age as a moderator (Farrell et al. 2018). Given that animal research (Ganella and Kim 2014; Kim and Richardson 2010) and threat-conditioning studies with humans (Waters et al. 2017) suggest that adolescence is a developmental period marked by impaired extinction learning relative to younger children and adults, further work is required to determine whether children and adolescents respond differently to strategies that target extinction mechanisms. The majority of key optimisation strategies identified within the adult literature (e.g. occasional reinforced extinction (Salkovskis et al. 2007), stimulus variability (Culver et al. 2012; Kircanski et al. 2012b) and affect labelling (Kircanski et al. 2012a; Niles et al. 2015) have yet to be explored with children and young people, highlighting further gaps in the evidence base. Finally, there were a limited number of studies and many provided insufficient data to calculate an effect size or explore potential moderators of immediate treatment outcomes or the association between exposure strategies and outcomes, such as the amount of time spent on exposure within treatment, how exposure was conducted (e.g. in vivo or imaginal), a focus on different disorders, or the amount or nature of parent involvement within treatment.

### Clinical Implications

Given the lack of replication of findings, any implications for how exposure may be carried out to achieve the best clinical effects must be extremely tentative at this stage and further research is required to be able to make any strong recommendations. However, the preliminary findings suggest that, during exposure, clinicians may find it beneficial to (i) ensure that the young person is engaged and able to master the information and skills, (ii) focus on the reduction of safety behaviours, (iii) ensure that both they and the parent/carer discourage the young person’s avoidance (iv) encourage the young person to do ‘difficult’ exposures within and between exposure sessions, (v) look for variable levels of fear within-exposure sessions such as greater emotional ups and downs, (vi) not try to do too many exposure exercises within the session (perhaps aiming for quality rather than quantity), and (vii) encourage the young person to discuss and process the experience following exposure.

### Future Research Directions

An important first step in future research will be to develop and agree validated measures of potential exposure optimisation variables that can be used within methodologically robust experimental and naturalistic studies. Future research should also address potential moderating factors; for example, examining the effect of strategies among specific age groups especially in light of the existing animal research (Ganella and Kim 2014; Shechner et al. 2014) and different disorders (e.g. Peterman et al. 2016). Finally, the studies in this review predominantly included clinical samples. In line with adult research, it may be more efficient for future research to examine targeted, theoretically-driven strategies in methodologically robust, preclinical studies, and use the findings from these studies to guide and prioritise the development of clinical research.

## Conclusion

Given that exposure appears to be the key ingredient in the treatment of anxiety disorders in children and young people (Ale et al. 2015; Kendall et al. 2005; Whiteside et al. 2015, 2019), it is critical that we understand how best to deliver it to improve treatment outcomes. This review identified a lack of consistent support for any potential optimisation strategies, wide ranging methodological inconsistencies among studies, and highlighted that most of the potential optimisation strategies identified within the adult literature have not been explored. Going forwards, future research should use consistent conceptualisations, methodological approaches, and outcome measures to enable meaningful comparisons between studies, examine other factors that have been found to facilitate exposure with adults, explore developmental differences (for example, between children and adolescents), and look to expand the research field by robust examination of theoretically-driven potential optimisation strategies.

## Footnotes

1 The Diagnostic and Statistical Manual Fifth Edition (DSM-5) (American Psychiatric Association 2013) reclassified obsessive compulsive disorder (OCD) and post-traumatic stress disorder (PTSD) from “anxiety disorders” to other categories. However, anxiety disorders, OCD and PTSD are all characterised by clinically significant fear, anxiety and distress in response to stimuli and/or situational cues (McGuire et al. 2016). As such, these disorders have been referred to as ‘anxiety disorders’ from here on.

2 The ‘2′ is a proximity search which specifies that the search terms (i.e. stress and disorder) are a maximum of two words apart from one another, regardless of the order in which they appear.
